# BART'S SYNDROME

**DOI:** 10.4103/0019-5154.41655

**Published:** 2008

**Authors:** Aruna Rajpal, R Mishra, K Hajirnis, M Shah, N Nagpur

**Affiliations:** *From the Department of Dermatology and Venerology, KJ Somaiya Medical College and Research Centre, Mumbai, India*; 1 *From the Department of Pathology, KJ Somaiya Medical College and Research Centre, Mumbai, India*; 2 *From the Department of Pediatrics, KJ Somaiya Medical College and Research Centre, Mumbai, India*

**Keywords:** *Bart's syndrome*, *congenital absence of skin*

## Abstract

A new-born girl presented with congenital absence of skin on the right leg and nail abnormalities. On second day of life, she developed multiple blistering skin lesions and died on seventeenth day of life. A positive family history of two other siblings, one male and one female who had blistering skin lesions and died within one and a half month of birth, was present. The diagnosis of Bart's syndrome was made on clinical presentation, family history and skin biopsy.

## Introduction

Bart's syndrome was described in a large family in 1966 and consisted of any one or a combination of the following three characteristics: congenital absence of skin, blistering and associated nail abnormalities. Congenital absence of skin is regarded now as a manifestation of epidermolysis bullosa (EB). Bart is not a disorder but a clinical sign seen in many forms of EB. Here we report a case of Bart's syndrome, an exceedingly rare disorder.

## Case History

**Fig. 1:**
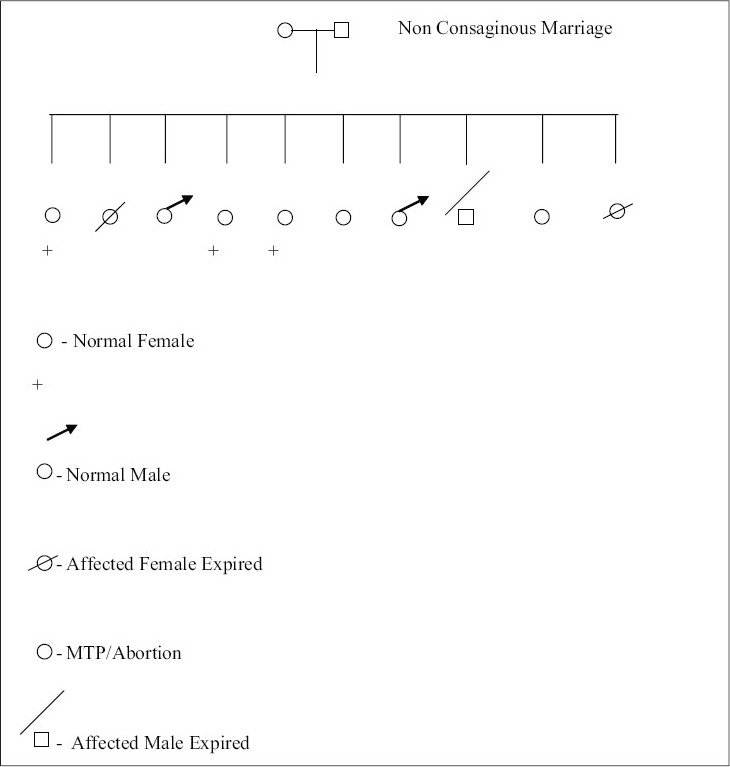
Pedigree

A new-born girl was referred to us for congenital absence of skin. She was the tenth child of a non-consanguinous couple. The pregnancy and delivery was normal. Both parents were apparently healthy and had no abnormalities of skin, skin appendages or mucous membrane. There was a positive family history of one female and one male child having blistering skin lesions, both of whom died within a few days of birth (Fig. 1).

**Fig. 2:**
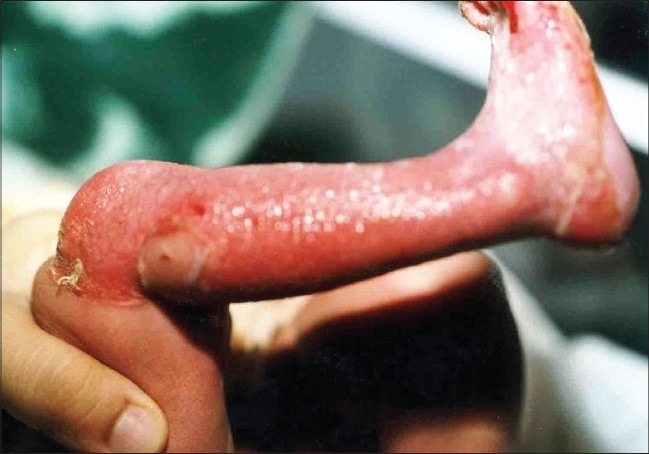
Absence of skin on right leg and right foot

**Fig. 3:**
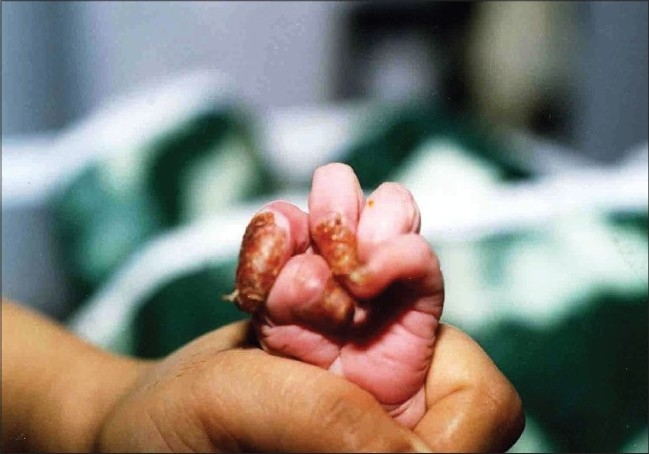
Erosions on distance phalanges of left hand

**Fig. 4:**
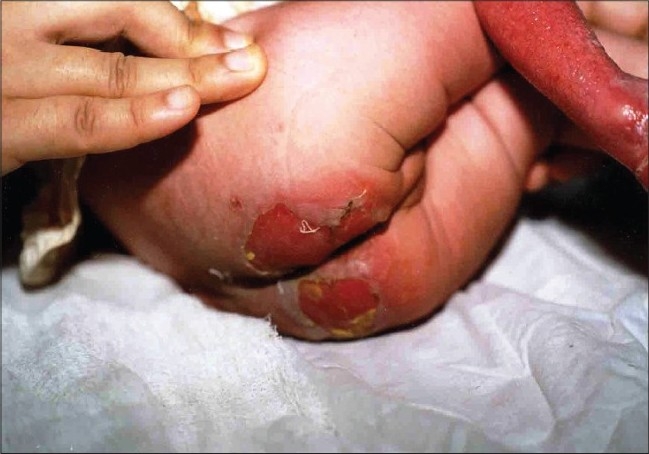
Erosion on buttocks

On examination of the child, there was absence of skin on the anterior and lateral surface of the right leg and dorsum of the right foot (Fig. 2). The defect extended proximally up to the knee and distally up to lateral half of sole. Erosions were present on distal phalanges of left hand (Fig. 3), fourth finger nail of left hand and big right toe nail. On the second day, the child developed blisters on the right shoulder, buttocks (Fig. 4), left elbow and scalp. There were no oral erosions. Systemic examination was normal.

**Fig. 5:**
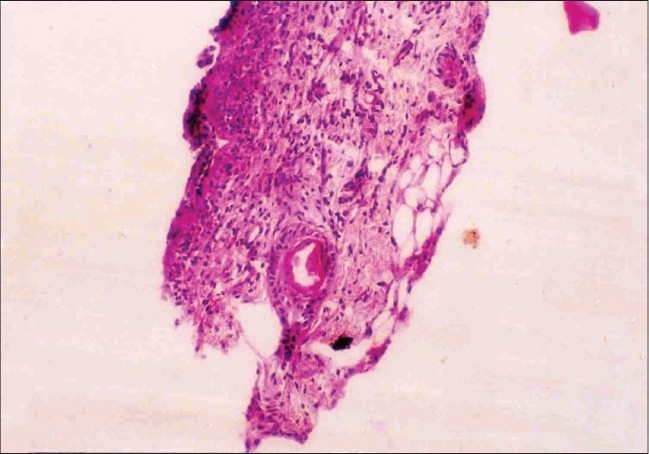
Skin biopsy from ulcerated skin lesion showing an ulcerated epidermis and hair follicle in upper dermis (H and E, ×2)

**Fig. 6:**
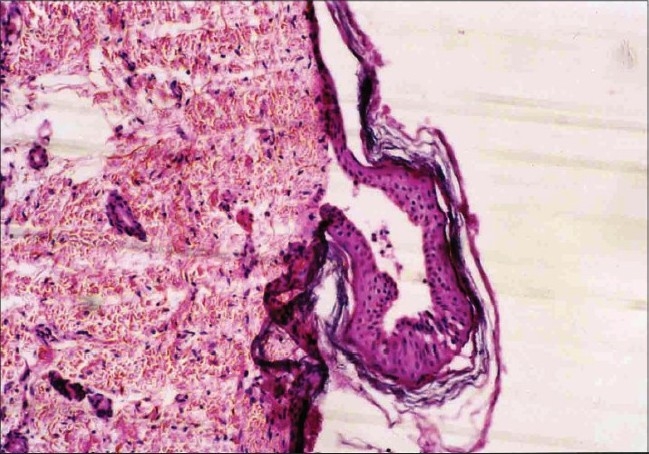
Skin biopsy from blister reveals a sub-epidermal blister (H and E, ×10)

**Fig. 7:**
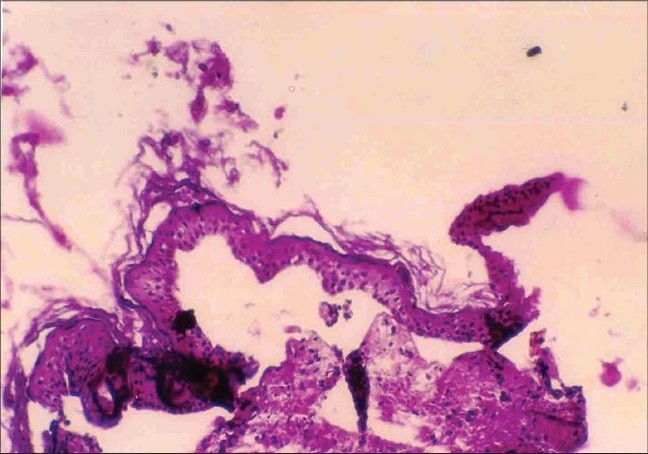
PAS stain reveals basement membrane along with dermis (PAS, ×10)

Skin biopsy from ulcerated skin lesions revealed ulcerated epidermis. Upper dermis showed neutrophils, eosinophils and an occasional hair follicle (Fig. 5). Skin biopsy from the blister showed a sub-epidermal blister (Fig. 6); PAS stain revealed basement membrane along with dermis (Fig. 7) suggesting the diagnosis of EB simplex or junctional EB. Electron microscopic studies could not be done due to financial constraints.

The child was discharged on request within a week. A subsequent home visit revealed that the child had died within three weeks of birth. The exact cause of death could not be determined in all three affected babies.

## Discussion

Bart *et al.*^1^ in 1966 reported a family with congenital absence of skin on the lower leg and widespread blistering of skin, mucous membrane and nail dystrophy. Twentysix members were affected in the family and penetrance was complete; father-son transmission was noted. Clinical findings suggested that the Bart syndrome may represent any of the three subtypes of EB: epidermal, junctional or dermal. Because no histologic, immune techniques, ultrastructural studies or genetic linkage methods were done in Bart's kindred, classification of this syndrome remained uncertain.

Kanzler *et al.*^2^ described a family in which persons in four generations had EB simplex with congenital absence of skin. They suggested that the family reported by Bart *et al.* probably also had generalized EB simplex of Koebner type. Their patient was the first to undergo electromicroscopy and immunofluoroscent mapping of both areas of skin fragility and congenital localized absence of skin. Electron microscopy and immunofluoroscent findings were identical at both the sites. These findings suggested ultrastructurally that the area of congenital absence of skin has the same pathogenic changes as those seen with EB. They pointed out that congenital absence of skin had been associated with all major types of inherited EB. According to them, localized absence of skin is a clinical manifestation of EB *in utero* and not a separate entity such as aplasia cutis congenita.

Zelickson *et al.*^3^ analysed Bart's kindred and demonstrated poorly formed anchoring fibrils and cleavage below the lamina densa on ultrastructural analysis. Genetic studies located the gene to chromosome 3p at or near the site of the gene and encoding for type VII collagen (COL7A1).

Christiano *et al.*^4^ performed mutation analysis in this family by DNA sequencing, which resulted in glycine to arginine substitution within the triple helical domain of type VII collagen in affected individuals.

Original idea that the absence of skin at birth indicates a specific form of EB has given way to the view that it a non-specific form of EB.

Lesions in Bart syndrome appear on extremities as well-defined, glistening red ulceration on dorsal and median aspect of foot, often extending up to the shin. They may be unilateral or less frequently bilateral, and probably results from rubbing one shin and foot with the other heel, perhaps in response to pruritis.

Mc Kinster *et al.*^5^ suggested that congenital absence of skin in Bart's syndrome may follow the lines of Blaschko. They reported six cases in which they observed bilateral and symmetric distribution of denuded area in an S-shaped broad band with sharply demarcated borders, involvement of toe webs and frequent involvement of soles. They felt that physical trauma *in utero* was too simplistic an explanation for the defect. This remains a debatable issue.

The skin biopsy of ulcerated lesion of our patient revealed an occasional hair follicle, thus ruling out aplasia cutis congenita. Since the PAS positive basement membrane zone remained with the dermis, EB dystrophica was ruled out. The pattern of inheritance in our case seems to be autosomal dominant in view of the fact that three neonates were affected and were of either sex. The histopathological feature and pattern of inheritance suggest that the child could have had EB simplex.

The Koebner, Weber Cockayne and Dowling Meara forms of EB simplex have rarely been associated with Bart's syndrome.^6^ There was a possibility that the child could have had junctional EB. The involvement of buttocks and paronychial inflammation is seen frequently in junctional EB.^7^ These clinical features along with the histopathology (PAS positive basement membrane zone with dermis) favour a diagnosis of junctional EB. But, a definitive diagnosis could have been made only on electron microscopy, which was not possible in our case. There has been association of Bart syndrome with Pyloric atresia.^8^,^9^ The possible cause of sudden death in our case could have been metabolic in origin like hypothermia and hypoglycemia due to congenital absence of skin or intercurrent infection.

Genetic counseling for this rare familial disorder is extremely important for affected families. DNA-based prenatal diagnosis using Chorionic villus sampling or Aminocentesis is available for junctional and dystrophic forms of EB, wherein the mutations have been characterized. Future avenues are currently under investigation for early prenatal diagnosis, including preimplantation genetics.
